# Population genetic structures of two ecologically distinct species *Betula platyphylla* and *B. ermanii* inferred based on nuclear and chloroplast DNA markers

**DOI:** 10.1002/ece3.5643

**Published:** 2019-09-10

**Authors:** Hua‐Ying Wang, Xiao Yin, Dong‐Xu Yin, Lin Li, Hong‐Xing Xiao

**Affiliations:** ^1^ Key Laboratory of Molecular Epigenetics of Ministry of Education Northeast Normal University Changchun China

**Keywords:** birch, *G3PDH*, LGM, Northeast China, refuge

## Abstract

Climatic oscillations during the last glacial maximum (LGM) significantly affected the distribution patterns and genetic structure of extant plants. Northeast China (NEC) is a major biodiversity center in East Asia, and the influence of historical climate change on NEC populations is critical for understanding species responses to future climate change. However, only a few phylogeographic studies of cool temperate deciduous tree species have been conducted in the area, and results are inconsistent for species with different niches or distribution areas. We employed multiple chloroplast and nuclear markers to investigate the genetic structure of two ecologically contrasting species, *Betula platyphylla* and *B*. *ermanii*, in NEC. Rare haplotypes were identified in the chloroplast genome of these species, and both exhibited high levels of nucleotide diversity based on a fragment of the nuclear gene *G3PDH* and microsatellites. Moreover, significant phylogeographic structure was detected for *B. platyphylla*, suggesting that these populations had recolonized from independent glacial refuges, whereas no genetic structure was found for *B*. *ermanii*.

**OPEN RESEARCH BADGES:**

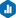

The nSSR datasets used in the current study and the table of pairwise FST (below diagonal) and its standardized F'ST (above diagonal) among 25 populations based on seven SSRs are available from the Dryad (DOI: https://doi.org/10.5061/dryad.230d176). Sequences generated from this study were deposited in GenBank under Accession nos. KY199568–KY200162 and MK819541–MK819970.

## Graphical abstract

Climatic oscillations during the Quaternary have led to the establishment of current terrestrial ecosystems. The high species richness and endemic species richness in the Changbai and Greater Khingan mountains suggest that these two regions may have different demographic histories. Here, phylogenetic analyses based on multiple chloroplast and nuclear genome markers demonstrated that populations of *B. platyphylla* in NEC were recolonized from two independent glacial refuges. More cold‐tolerant species was probably able to survive at higher latitudes, leading high levels of gene flow between the geographically separated populations during LGM, so no geographic structure was detected in *B. ermanii*.

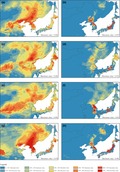

## 1 INTRODUCTION

Climatic oscillations during the Quaternary significantly affected the distribution patterns of animals and plants throughout the world, particularly in the Northern Hemisphere (Bytnerowicz, Omasa, & Paoletti, 2007; Davis & Shaw, 2001; Donoghue, Bell, & Li, 2001; Hewitt, 2004; Woillard, 1978). During the last glacial maximum (LGM), for example, species survived in glacial refuges and subsequently spread to suitable environments as the climate warmed, which eventually led to the establishment of the current terrestrial ecosystems (Normand et al., 2011; Qi et al., 2012; Willner, Pietro, & Bergmeier, 2009).

Persistent global diversity anomalies also indicate that species richness is controlled by past climate (McGlone, 1996; Ricklefs, 2004). Areas with a more stable climate or steep topographic relief permitted short‐distance climate tracking and resulted in high species richness (Jetz, Rahbek, & Colwell, 2004; Turner, 2004). Notably, the LGM climate constitutes the best single set of explanatory variables for range‐restricted species richness (Svenning & Skov, 2007), and globally, glacial refuges with generally high endemic species richness experienced little Quaternary climate change (Jansson, 2003). Given the high species richness and high number of endemic species and relict species, Wang and Liu (1994) proposed that southwestern China was a glacial refuge during the LGM.

To identify glacial refuge and recolonization routes during and after the LGM, a large number of investigations have been performed based on floristic, palynological, and population genetics data (Cartens, Brunsfeld, Demboski, Good, & Sullivan, 2005; Comes & Kadereit, 1998; Soltis, Gitzendanner, Strenge, & Soltis, 1997). Indeed, recent population genetic analyses from several independent studies have identified multiple refuges for angiosperms, such as the Hengduan, Tianmu, Qinling, Dabashan, and Taihang mountains in China (Chiang et al., 2006; Chou, Thomas, Ge, LePage, & Wang, 2011; Huang, Hwang, & Lin, 2002; Huang et al., 2010; Qiu, Fu, & Comes, 2011; Zhang, Yan, Zhang, & Zhou, 2008; Zhang, Zeng, Shan, & Ma, 2012; Zhang, Chiang, George, Liu, & Abbott, 2005) and the Baekdudaegan in North Korea and South Korea (Chung, López‐Pujol, & Chung, 2017; Chung, Son, et al., 2018; Chung, Vu, et al., 2018). Although tremendous progress has been achieved in recent decades, these studies have been concentrated in southern, southwestern, and northern China. In addition, studies on some widespread species, such as *Acer mono* var. *mono*, *Phellodendron amurense*, *Allium neriniflorum*, and *A. tubiflorum*, have indicated clear genetic differentiation between taxa from Northeast China (NEC) and other regions; thus, we speculate that NEC taxa constitute a relatively independent genetic clade (Liu et al., 2014; Yang et al., 2016; Yang, Zhou, Huang, & He, 2017).

**Figure 1:**
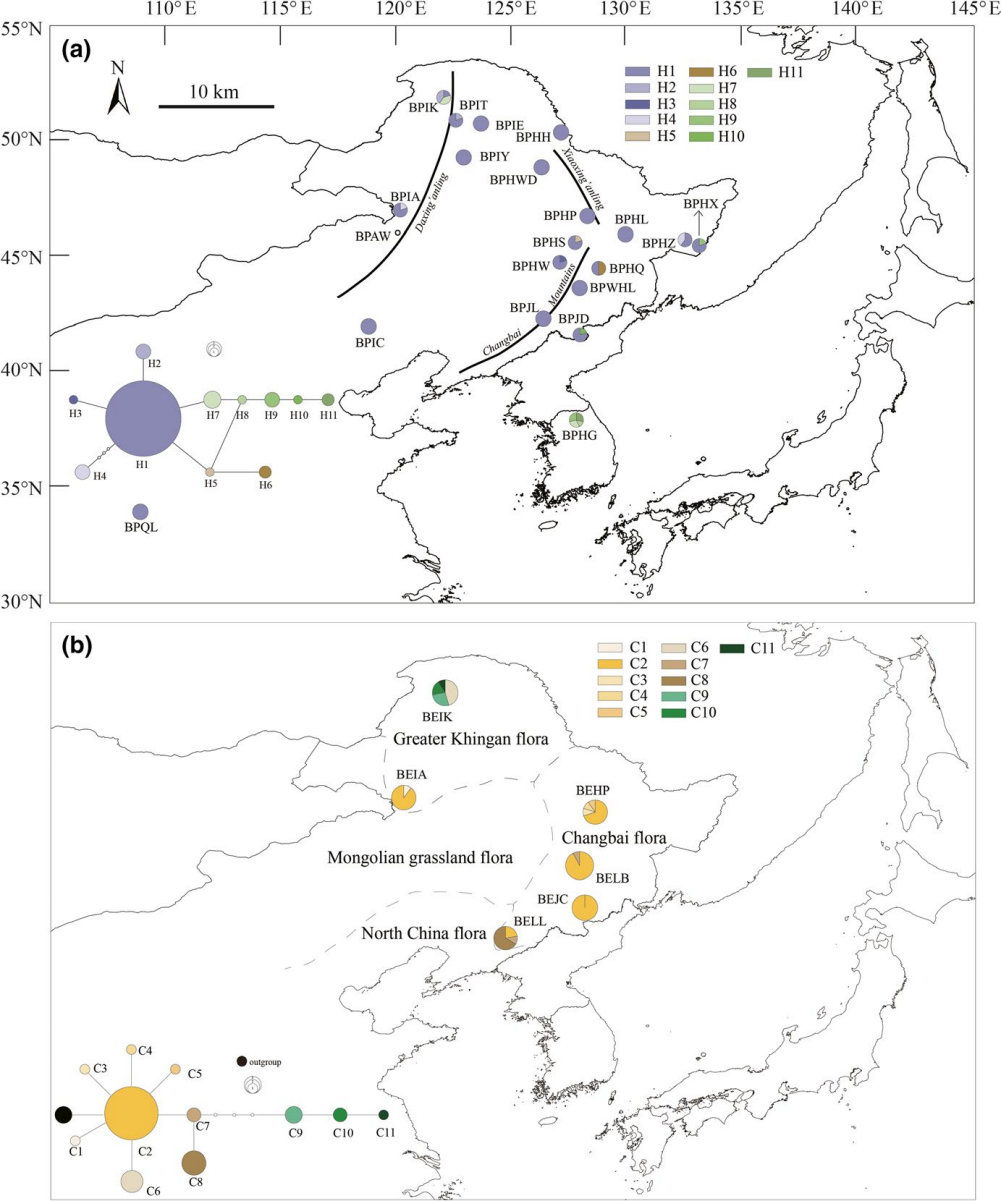
Sampling locations and parsimony networks of cpDNA for *Betula platyphylla* (a) and for *B. ermanii* (b). Each circle represents one haplotype, and the circle area is proportional to the frequency of each haplotype. The dots indicate missing haplotypes and the distribution of the four floras in Northeast China according to Fu (2003)

Northeast China is a megadiversity area with a complex topography including the Northeast Plain and major mountain ranges, such as the Changbai Mountains, Xiaoxing'anling Mountains, and Daxing'anling Mountains (China, 1998; Xu, Wang, & Xue, 1999) (Figure 1a). Controlled by the high‐latitude East Asia monsoon, the warm temperate climate of the south becomes a cooler temperate climate in the north. With these complex topographic and climatic gradients, NEC is a major biodiversity center in East Asia (Chen, 1998). The area has been divided into four distinct terrestrial ecosystems according to vegetation types, namely northern China flora, Changbai flora, Greater Khingan flora, and Mongolian grassland flora (Figure 1b) (Fu, 2003). The major Quaternary glaciation appears not to have occurred in NEC; however, the climate was at least 6–8°C cooler and more arid during the LGM than it is today (Sun & Chen, 1991).

Phylogeographic studies of taxa from NEC have proposed two views (Bai, Liao, & Zhang, 2010; Bao et al., 2015; Hu et al., 2008; Tian et al., 2009; Zeng, Wang, Liao, Wang, & Zhang, 2015). Based on analysis of the genetic structure of *Fraxinus mandshurica*, which is widely distributed in the Changbai Mountains and the southern part of the Xiaoxing'anling Mountains in NEC, Hu et al. (2008) concluded that a continuous glacial refuge may have existed in the Changbai Mountains (between 40°N and 42°N). In contrast, Bai et al. (2010) and Zeng et al. (2015) argued that the current plant communities of NEC were recolonized from several isolated refuges, as based on *Juglans mandshurica* and *Quercus mongolica* in the Changbai Mountains and the south of the Xiaoxing'anling Mountains, also with distribution in the Daxing'anling Mountains and north of the Xiaoxing'anling Mountains, respectively. Therefore, we cannot rule out possibility that taxa in the Daxing'anling Mountains or in the north of the Xiaoxing'anling Mountains had colonized from another refuge. However, as samples from the Daxing'anling Mountains in these studies were rare or absent, further studies focusing on NEC, especially the Daxing'anling Mountains, are necessary to obtain a full understanding of how current land ecosystems have formed. In addition, as *J. mandshurica* and *Q. mongolica* are more cold‐tolerant than *F. mandshurica*, we can infer that ecologically distinct species may have had divergent evolutionary histories during the LGM, resulting in different current distribution ranges.

Birch species are common and widely distributed in cool temperate and boreal forests across Eurasia. For *Betula*, phylogeographic studies have been confined to Europe and Japan, with a special emphasis on an LGM refuge that is farther north than initially thought (Binney et al., 2009; Eidesen, Alsos, & Brochmann, 2015; Lascoux, Pyhäjärvi, Källman, & Savolainen, 2008; Maliouchenko, Palmé, Buonamici, Vendramin, & Lascoux, 2007; Mee & Moore, 2014; Palmé, Su, Palsson, & Lascoux, 2004; Palmé, Su, Rautenberg, Manni, & Lascoux, 2003; Tsuda, Nakao, Ide, & Tsumura, 2015; Tsuda, Semerikov, Sebastiani, Vendramin, & Lascoux, 2016; Willis, Rudner, & Sümegi, 2000). In NEC, *Betula ermanii* Chamiss is restricted to high‐elevation habitats and represents the upper limit of the tree line. Among other congeners in NEC, *B. platyphylla* Sukaczev (2*x* = 28) has almost the same distribution as *B. ermanii* (4*x* = 56), though they have different ploidy levels. *B. platyphylla* is a pioneer species that is widely distributed in low‐elevation regions and is usually a dominant species in forest systems. *B. ermanii* and *B. platyphylla* are all distributed from Siberia to Far East Asia, and NEC comprises the main and central range of the two species according Tsuda et al. (2016). These species belong to different subsets based on morphological features, but some sharing of cpDNA haplotypes has been detected (Tsuda & Ide, 2010). Moreover, STRUCTURE results indicate some admixture for the two species based on seven nuclear simple sequence repeats (nSSRs) (figure 1 in Tsuda et al., 2016), and the pairwise *F*_ST_ or *F*'_ST_ between them is low (table 2 in Tsuda et al., 2016). Therefore, the two birch species are ideal for addressing how differences in ecological tolerance or niche can potentially lead to divergent demographic histories and differences in population structure.

To obtain the genetic structures of the two congeneric species, we surveyed nucleotide variation patterns of chloroplast genomes for both species. As the chloroplast genome is maternally inherited and usually shows a low mutation rate, we also sequenced a fragment of the single‐copy nuclear gene glycerol‐3‐phosphate dehydrogenase (*G3PDH*) and 10 nSSRs. The aims of the study were to (a) evaluate the population genetic structure of the two birch species and (b) assess whether these species in NEC survived in multiple refuges during the LGM. The findings of our study may provide insight into the demographic history of ecological systems in NEC.

## 2 MATERIALS AND METHODS

### 2.1 Sample collection and DNA extraction

Leaf material of *B*. *platyphylla* and *B. ermanii* was collected from 19 and 6 locations across NEC, amounting to a sample size of 174 and 136 individuals, respectively (Figure 1, Table S1). In addition to 19 populations in NEC, another 8 individuals of *B. platyphylla* were collected from the Qinling Mountains. Of the 19 *B. platyphylla* populations, 5 populations were obtained from the Changbai Mountains, 5 populations from the Xiaoxing'anling Mountains and 9 populations from the Daxing'anling Mountains (Table S1). We sampled 3, 1, and 2 populations of *B. ermanii* from the Changbai Mountains, Xiaoxing'anling Mountains, and Daxing'anling Mountains, respectively (Table S1). To avoid collecting clones, all these individuals were at least 100 m apart from each other; the material was dried with silica gel. In China, no specific permission is required for the collection of specimens of *B. platyphylla* or *B. ermanii*, which are widely distributed in NC and NEC, respectively. Fourteen samples of *B. platyphylla* were kindly provided by Herbarium Hallym University (HHU).

We undertook the formal identification of the plant material used in the study, and voucher specimens were collected for each population and deposited with Northeast Normal University Herbarium (NENU4081‐NENU4101 for *B. platyphylla* and NENU4102‐NENU4107 for *B. ermanii*). Total genomic DNA was extracted from dried leaves for each individual of *B. ermanii* using the Plant Genomic DNA kit (TianGen). In contrast, as the quality of *B. platyphylla* DNA extracted by the Plant Genomic DNA kit was poor, we used a modified CTAB method to extract genomic DNA from this species (Doyle, 1987).

### 2.2 Microsatellite genotyping

Microsatellite genotyping was conducted for *B. platyphylla* and *B. ermanii* using 10 primer pairs (Table 1). All primer sequences were obtained from previous studies (Kulju, Pekkinen, & Varvio, 2004; Ogyu, Tsuda, Sugaya, Yoshimaru, & Ide, 2003; Wu, Lian, & Hogetsu, 2002). Seven of these primer pairs (*BP4*, *BP12*, *BP15*, *BP16*, *BP17*, *BE6*, and *BE12*) were employed to analyze the genotypes of both species. Of the remaining three microsatellites, one (*BE8*) was scored for *B. platyphylla*, and two (*BP10* and *BE7*) were genotyped for *B. ermanii*. PCR amplifications using these nSSR primers were performed in a 20‐μL reaction volume including 50 ng of genomic DNA, 1 × PCR buffer (plus Mg^2+^), 0.2 mM of each dNTP, and 0.5 μM of each primer, with each forward primer labeled with fluorescent dye (FAM, TAMRA, or HEX) (Invitrogen) and 1 unit (U) of *Taq* polymerase (Takara). The PCR amplification reactions were carried out using an ABI2720 Thermocycler (Applied Biosystems) according to the following procedure: an initial denaturation step at 95°C for 5 min, followed by 35 cycles of 30 s at 94°C, 30 s at an optimal annealing temperature, and 30 s at 72°C, and a final elongation step at 72°C for 8 min. The amplified fragments were mixed and resolved using an ABI 3730 DNA sequence (Applied Biosystems) capillary electrophoresis instrument. We then determined genotypes based on the dosage of PCR product peaks (Tsuda et al., 2016) and carefully checked each locus to avoid errors. However, for some individuals, especially tetraploids, of *B. ermanii*, the peaks were insufficiently clear to determine allele copy number in this way.

**Table 1:** Primer sequences and some characteristics of the microsatellite loci

Locus	Primer sequence	*N*_A_	*Betula platyphylla*		*N*_A_	*Betula ermanii*		References
			*h*	Ar		*h*	Ar	
BP4	F: GGCAACCAGCAGCAATCTGAC	17	0.747	4.528	11	0.785	8.021	WU et al. (2002)
	R: ATGCCCAAGGACGACTAGACC							
BP10	F: GTTGTAATGCAAACACATGGG	–	–	–	15	0.655	8.165	WU et al. (2002)
	R: TCTGTGTCATAATTGGGTAGG							
BP12	F: GCCTGCTTTCCATTCGTACAC	4	0.211	1.683	15	0.863	10.534	WU et al. (2002)
	R: TCCCGGTTAAGTCAAAGTTCC							
BP15	F: ACGCTTTCTTGATGTCAGCC	20	0.874	5.549	19	0.815	10.300	Kulju et al. (2004)
	R: TCACCAAGTTCCTGGTGGAT							
BP16	F: CAGTGTTTGGACGGTGAGAA	12	0.589	3.356	10	0.700	6.609	Kulju et al. (2004)
	R: CGGGTGAAGTAGACGGAACT							
BP17	F: AAGGGCACCTGCAGATTAGA	20	0.812	4.717	5	0.093	2.945	Kulju et al. (2004)
	R: AAAATTGCAACAAAACGTGC							
BE6	F: GTTGCTGCTCACCTCAAAAATGT	20	0.782	4.286	19	0.832	11.897	Ogyu et al. (2003)
	R: TGCACGGTTGGAGAATAGAAGAA							
BE7	F: CGAAACCCTAAACCCCTCCT	–	–	–	15	0.810	10.429	Ogyu et al. (2003)
	R: AAACCGTACACCTAAACCAA							
BE8	F: GTCAGGTAGTTAGGGGCATT	18	0.814	4.951	–	–	–	Ogyu et al. (2003)
	R: AAGCGGGTAAAAGGAGTGTG							
BE12	F: ATCTCCTCTGCTTCTTCACA	9	0.736	3.850	19	0.880	12.452	Ogyu et al. (2003)
	R: ATCTCACACCTCCACTCCTC							

Abbreviations: Ar, allelic richness; *h*, gene diversity; *N*_A_, number of alleles.

### 2.3 Chloroplast and nuclear gene sequencing

We examined polymorphisms in the chloroplast genomes of the two *Betula* species using 40 universal primer pairs (Table S2). For each primer pair, we sequenced three individuals of each species from geographically isolated populations. No nucleotide polymorphisms were detected in *B. platyphylla*. In contrast, a few variable sites were identified in three cpDNA regions (*trnL‐F‐trnL‐C*, *trnH‐psbA*, and *psbk‐psbI*) for *B. ermanii*. The three cpDNA regions were then sequenced in subsets of 98 and 64 individuals of *B. platyphylla* and *B. ermanii*, respectively. Each individual of three species, *B. dahurica* Pallas, *B. costata* Trautvetter, and *B. ovalifolia* Ruprecht, was also sequenced as outgroups. Polymerase chain reaction (PCR) for the three chloroplast regions was performed using a 30‐μl reaction containing 1 × PCR buffer (plus Mg^2+^), 0.2 mM of each dNTP, 0.5 μM of forward and reverse primers, 1 U of *Taq* DNA polymerase (Takara), and 50 ng of genomic DNA. The PCR protocol consisted of 4 min of initial denaturation at 94°C, 35 cycles of denaturation for 45 s at 94°C, annealing at *T*_m_ for 30 s (Table S2), and extension for 90 s at 72°C, and a final extension at 72°C for 8 min. PCR was performed using an ABI2720 Thermocycler (Applied Biosystems). The PCR products were verified by 1.5% agarose gel electrophoresis and sequenced using an ABI‐3730 analyzer (Applied Biosystems).

Because low nucleotide polymorphisms were observed across the chloroplast regions, we designed a primer targeting the nuclear single‐copy gene glyceraldehyde‐3‐phosphate dehydrogenase (*G3PDH*, GenBank No.: KM586058). The selected nuclear gene was successfully amplified across all populations of *B. platyphylla* and *B. ermanii* in a 20‐μl reaction using an ABI2720 Thermocycler (Applied Biosystems) under the following PCR protocol: 4 min of initial denaturation at 94°C, followed by 35 cycles of denaturation for 30 s at 94°C, annealing at 68°C for 30 s, and extension for 90 s at 72°C, ending with a final extension step at 72°C for 8 min. The PCR products were ligated to a pMD‐18 vector (Takara) and transformed into the competent *Escherichia coli* DH5α cells (Takara) according to the manufacturer's instructions. Positive clones were validated using M13 primers (M13F: 5′‐GTAAAACGACGGCCAG‐3′, M13R: 5′‐CAGGAAACAGCTATGAC‐3′). An average of one and four clones for each individual of *B. platyphylla* and *B. ermanii* were sequenced, and a total of 179 and 351 positive clones were sequenced for *B. platyphylla* and *B. ermanii* using an ABI 3730 DNA analyzer (ABI), respectively (Table S1).

### 2.4 Microsatellite data analysis

The number of alleles (*N*_A_), genetic diversity (*h*) (Nei, 1987a), proportion of private alleles (*N*_P_), allelic richness (Ar) (El Mousadik & Petit, 1996), fixation index (FIS), deviation from zero indicated significant deviations from Hardy–Weinberg equilibrium (HWE), were calculated based on SSR markers using FSTAT software (Goudet, 1995). To compare genetic diversity and differentiation (*F*_ST_), tetraploid individuals were regarded as two diploid individuals using FSTAT software. This approach was employed in previous studies (Tsuda et al., 2016), and it demonstrated that the biases caused by duplication of the sample size in tetraploid species can be ignored. Moreover, observed heterozygosity (Ho), total heterozygosity (Ht), the standardized value of *F*_ST_ (*F*'_ST_), and principal component analysis (PCA) were performed using GenoDive (Meirmans & Van Tienderen, 2004). Analysis of molecular variance (AMOVA, Excoffier, Smouse, & Quattro, 1992) was employed to evaluate how genetic variability is structured in different groups of these two species. When analysis was performed on a total of 315 individuals, according to Tsuda et al. (2016), the genotypes of *B. platyphylla* individuals were duplicated to show tetraploid‐like genotypes using GenoDive software, which is not able to analyze samples of different ploidy levels.

The population genetic structure of the total samples and each species was analyzed using the Bayesian clustering program STRUCTURE version 2.3.3 (Pritchard, Stephens, & Donnelly, 2000). This program estimates the number of genetic clusters (*K*) within each species without consideration of sampling location. In genetic structure analysis of total samples, the genotypes of the *B. platyphylla* individuals were duplicated to show tetraploid‐like genotypes. As ambiguous locus was found for a few individuals of *B. ermani*, we created another dataset of *B. ermanii* for STRUCTURE analysis according Hu et al. (2019). In the new dataset, we used missing data (−9) to replace the repeated genotypes when a tetraploid has two or three alleles. For example, if a tetraploid has three alleles, such as AABC, ABBC, or ABCC, we used ABC‐9; if it has two alleles, such as ABBB, AAAB, or AABB, we encoded it as AB‐9‐9. To obtain the best *K* value for each species, we preassigned the number of *K* ranging from one to the number of populations for each subset with ten independent replicates. Each run was performed with 100,000 MCMC iterations and an initial burn‐in of 100,000, as based on the admixture model and the correlated allele frequencies model. The final posterior probability of *K*, ln *p*(*K*), and Delta *K* (Δ*K*) was calculated using STRUCTURE HARVESTER (Earl & vonHoldt, 2012) to determine the most likely *K* value, as suggested by Pritchard et al. (2000) and Evanno, Regnaut, and Goudet (2005). Replicate runs were summarized using CLUMPP (Jakobsson & Rosenberg, 2007) software and visualized using DISTRUCT (Rosenberg, 2004) software.

### 2.5 Nucleotide polymorphism and phylogenetic analysis

DNA sequences of both chloroplasts and nuclear genes were aligned using the software Clustal X version 1.81 (Thompson, Gibson, Plewniak, Jeanmougin, & Higgins, 1997) and adjusted and edited using BioEdit v7.1.3.0 if necessary (Hall, 1999). The genetic diversities of the two species were calculated using the program DnaSP v5 (Librado & Rozas, 2009), including the haplotype diversity (Hd), nucleotide diversity (*π*) (Nei, 1987b) and (*θ*) (Watterson, 1975), segregating sites (*S*), and number of haplotypes (*h*), with *p* values based on the chi‐squared text or *t* test (two‐tailed). In addition, the neutrality test statistics Tajima (1983) and Fu and Li (1993) were estimated for cpDNA and nuclear genes using DnaSP (Librado & Rozas, 2009). Relationships of the haplotypes of chloroplast genes obtained were joined and analyzed with statistical parsimony networks using TCS 2.1 (Clement, Posada, & Crandall, 2000), with a 95% connection limit for cpDNA, and indels were coded as the 5th state. The unprocessed networks produced by TCS were redrawn using the vector drawing software Adobe Illustrator CS5 (Adobe Systems Inc.). In addition, the population differentiation of the two species was evaluated in terms of both unordered alleles (GST) and ordered alleles (NST) using Permut software (Pons & Petit, 1996) with 1,000 random haplotype permutations. ML trees based on *G3PDH* were generated using RAxML (Stamatakis, 2014) with the GTRGAMMA model and 1,000 bootstrap replicates.

### 2.6 Ecological attributes and ecological niche modeling

We used the maximum entropy method in MAXENT v3.3.3k (Phillips, Anderson, & Schapire, 2006) to infer the possible distributions of *B. platyphylla* and *B. ermanii* at present and during the LGM and last interglacial (LIG). In addition to our sample locations, the geographic locations of all *B. platyphylla* and *B. ermanii* populations were retrieved from Chinese Virtual Herbarium (http://www.cvh.org.cn/) and Global Biodiversity Information Facility (GBIF) (http://www.gbif.org). We also removed duplicate records within 5 km (a spatial resolution of 2.5 arc‐min) of one another to reduce the effect of spatial autocorrelation in climate variables, resulting in a total of 436 and 228 occurrence records covering the current distribution ranges of *B. platyphylla* and *B. ermanii*, respectively. For ecological niche modeling, all 19 climatic variables were downloaded from the WorldClim database (http://www.worldclim.com/) at 2.5‐arc‐min grids for both the present and LGM (approx. 21 Ka BP) and LIG (approx. 130 Ka BP) at 30‐s arc‐sec.

We grouped bioclimatic variables with strong collinearity (i.e., Pearson's correlation coefficient *r* ≥ |.80|) (Chung, Son, et al., 2018; Chung, Vu, et al., 2018). The variables from these groups were then selected based on their relative contribution to the model (percent contribution, jackknife tests of variable importance) and the shape of their response curves. Four variables were selected: annual mean temperature (bio1), minimum temperature of the coldest month (bio6), mean temperature of the coldest quarter (bio11), precipitation of the warmest quarter (bio18) for *B. platyphylla*, and bio1, bio6, bio11, and annual precipitation (bio12) for *B. ermanii*. We then ran 5,000 iterations for the data with 15 replicates, of which 40% of the data were used to simulate the model and the remaining 60% to test the model. The Climate System Model (CCSM) (Collins et al., 2006) and Interdisciplinary Research on Climate Model (MIROC) (Hasumi & Emori, 2004) were used to infer the extent of suitable habitat during the LGM.

## 3 RESULTS

### 3.1 Population genetic structure based on nSSR markers

**Figure 2:**
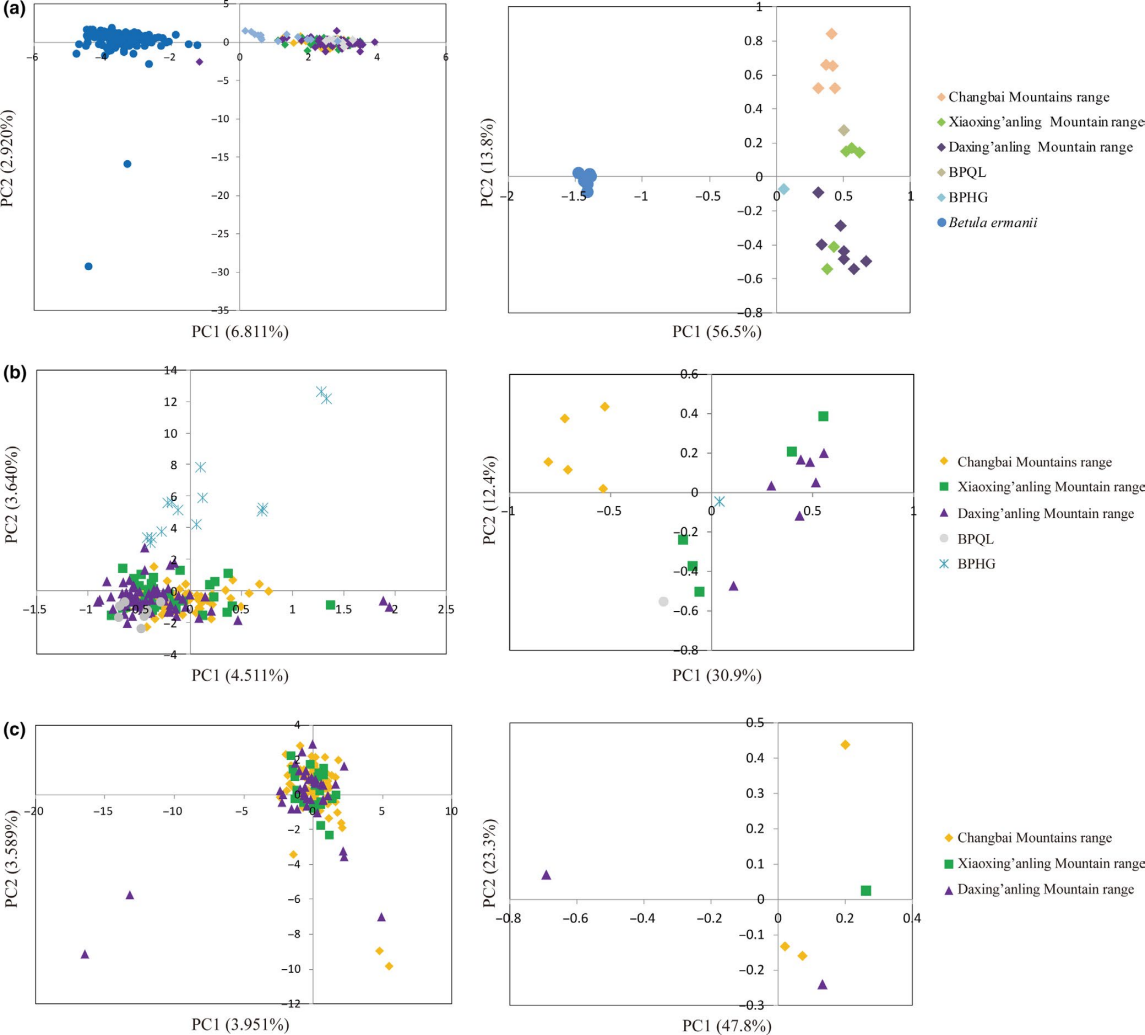
Principal component analysis of total populations (a), *Betula platyphylla* (b) and *B. ermanii* (c). The left is the result of PCA analysis in individual‐level, and the right is in population level of each taxa

**Figure 3:**
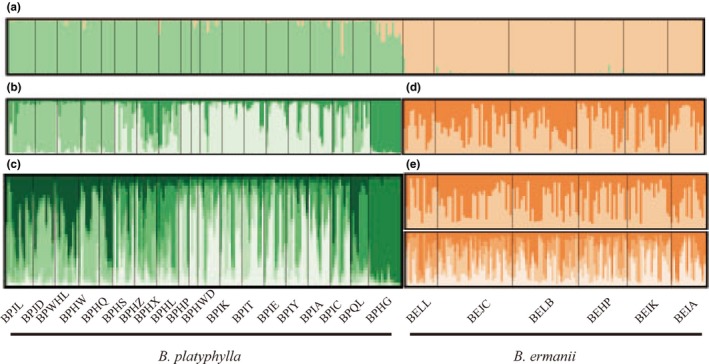
Results of Bayesian cluster analysis of two species at *K* = 2 (a), *Betula platyphylla* populations at *K* = 3 (b) and *K* = 7 (c) based on the Δ*K* and ln *p*(*K*) values, *B. ermanii* dataset with our manual estimates of allele copy numbers at *K* = 2 (d) and *B. ermanii* dataset with missing data at *K* = 2 (top) and *K* = 4 (e). Populations are separated by black bars and identified at the bottom

Both individual‐based and population‐based PCA revealed a separate pattern of genetic structure between the two species (Figure 2a), though some admixture was shown in the STRUCTURE results (Figure 3a). The aforementioned observations were confirmed by AMOVA (Table S3), and the proportion of genetic variance partitioned between the species was 38.5% (*ρ*_CT_ = 0.385). Higher differentiation was detected between populations of *B. platyphylla* compared with *B. ermanii*, as revealed by pairwise *F*_ST_ and *F'*_ST_ (online data).

PCA analysis based on nine nSSR markers showed a clear spatial genetic structure between different geographic regions for *B. platyphylla* (Figure 2b), especially in population‐based PCA, and STRUCTURE analysis revealed a genetic structure similar to that detected by PCA. The *B. platyphylla* populations were divided into three clusters according to the highest Δ*K* (Figure S1). When *K* = 3 (Figure 3b), three genetic clusters were observed, one of which was composed of five Changbai Mountain populations, and another one of which included all populations from the Daxing'anling Mountains; the population from South Korea showed different genetic structures. In contrast, admixed assignments were found for the Xiaoxing'anling Mountain populations and Qingling Mountain populations. Although ln *p*(*K*) increased constantly from *K* = 1 to *K* = 7 (Figure S1), a clear spatial genetic structure was detected (Figure 3c). Nevertheless, no spatial genetic structure was observed for *B. ermanii* based on both datasets (Figure 3d,e).

### 3.2 Population genetic structure based on cpDNA and nuclear sequences

Of the three cpDNA regions, *trnL‐F‐trnL‐C*, *trnH‐psbA*, and *psbk‐psbI*, successful amplification was achieved for 98 and 64 samples of *B. platyphylla* and *B. ermanii*, and the combined sequences were 1,441 bp in length after trimming and alignment. Eleven variable regions were identified in the three cpDNA regions: 2 nucleotide substitutions and 9 indels and gaps, corresponding to 22 haplotypes (Table S4). Eleven haplotypes for cpDNA were detected in each species (Figure 1), with haplotypes H1 and C2 dominating in *B. platyphylla* and *B. ermanii* populations, respectively (Figure 1). Among populations of *B. platyphylla*, most were fixed for haplotype H1, though populations BPHG and BPIK exhibited three and four haplotypes, as sampled from South Korea and Daxing'anling Mountains, respectively. Most populations with two haplotypes were located in the Changbai Mountains and Daxing'anling Mountains.

However, in *B. ermanii* populations, only population BEJC was monomorphic for haplotype C2, and the other populations had multiple haplotypes. Haplotypes C9, C10, and C11, adjacent to three missing haplotypes, were rare and unique to the BEIK populations located in the Daxing'anling Mountains. A relatively high GST and NST (NST = 0.441, GST = 0.429) was found across *B. ermanii* populations, and NST was not significantly different from GST. However, NST (NST = 0.308) was significantly higher than GST (GST = 0.224) in *B. platyphylla* populations, indicating the presence of phylogeographical structure according to Pons and Petit (1996).

The ML tree topologies obtained for each species based on the *D3PDH* gene were highly similar, with both being divided into two main clades. For *B. platyphylla*, although the populations from South Korea and the Qingling Mountains are slightly different from the NEC populations, no obvious genetic breaks were found (Figure S2). Interestingly, in the two clades of *B. ermanii*, 64 of 97 individuals with multiple clones were polyphyletic (Figure S3).

### 3.3 Genetic diversity

Genetic diversity was estimated based on microsatellite data at both the population and locus level (Tables S1 and S5). At the population level, the values of *N*_A_, *N*_P_, Ar, Ho, and Ht ranged from 25 to 45, 0 to 0.062, 3 to 4.1, 0.391 to 0.688, and 0.562 to 0.0710 in *B. platyphylla* and from 47 to 68, 0.002 to 0.033, 7.11 to 8.56, 0.493 to 0.585, and 0.686 to 0.728 in *B. ermanii*, respectively (Table S1). Although *B. platyphylla* (*N*_A_ = 95) sampled in NEC presented almost the same number of alleles as *B. ermanii* (*N*_A_ = 100), the values of *N*_A_ for each *B. platyphylla* population were much lower than those for *B. ermanii*, as were the values of Ar and Ht (*t* test, *p* < .01). However, Ho values for *B. ermanii* populations were not different from those for *B. platyphylla* populations. Additionally, allelic diversity was highly variable across loci, excluding BP12, and *B. ermanii* populations exhibited higher genetic diversity at the remaining five loci (Table S5).

Based on the results of genetic structure, we categorized BPHWD and BPHH as belonging to the Daxing'anling Mountains, though these populations are located at the junction of the Daxing'anling Mountains and Xiaoxing'anling Mountains. Despite the similar genetic diversity exhibited in the three different geographic regions of each species, it is worth noting that the probability of private alleles was very low in the Xiaoxing'anling Mountains. Significant and positive FIS values were detected for both species, indicating a significant deficit of heterozygotes in the populations (Table S1).

With regard to the *G3PDH* gene fragment, DNA sequences of 179 *B. platyphylla* individuals generated an 862‐bp alignment yielding 282 variable sites and 154 haplotypes. The haplotype diversity of this gene was very high (Hd = 0.996) at the species level. Similar genetic diversity was also observed in the six populations of *B. ermanii*. A total of 351 sequences, corresponding to 258 haplotypes, generated a 904‐bp alignment with 296 polymorphic sites. There were no significant differences in the average nucleotide diversity of the populations from the three different geographic areas of both species (Table S1).

### 3.4 Current and past distribution of the two Betula species

**Figure 4:**
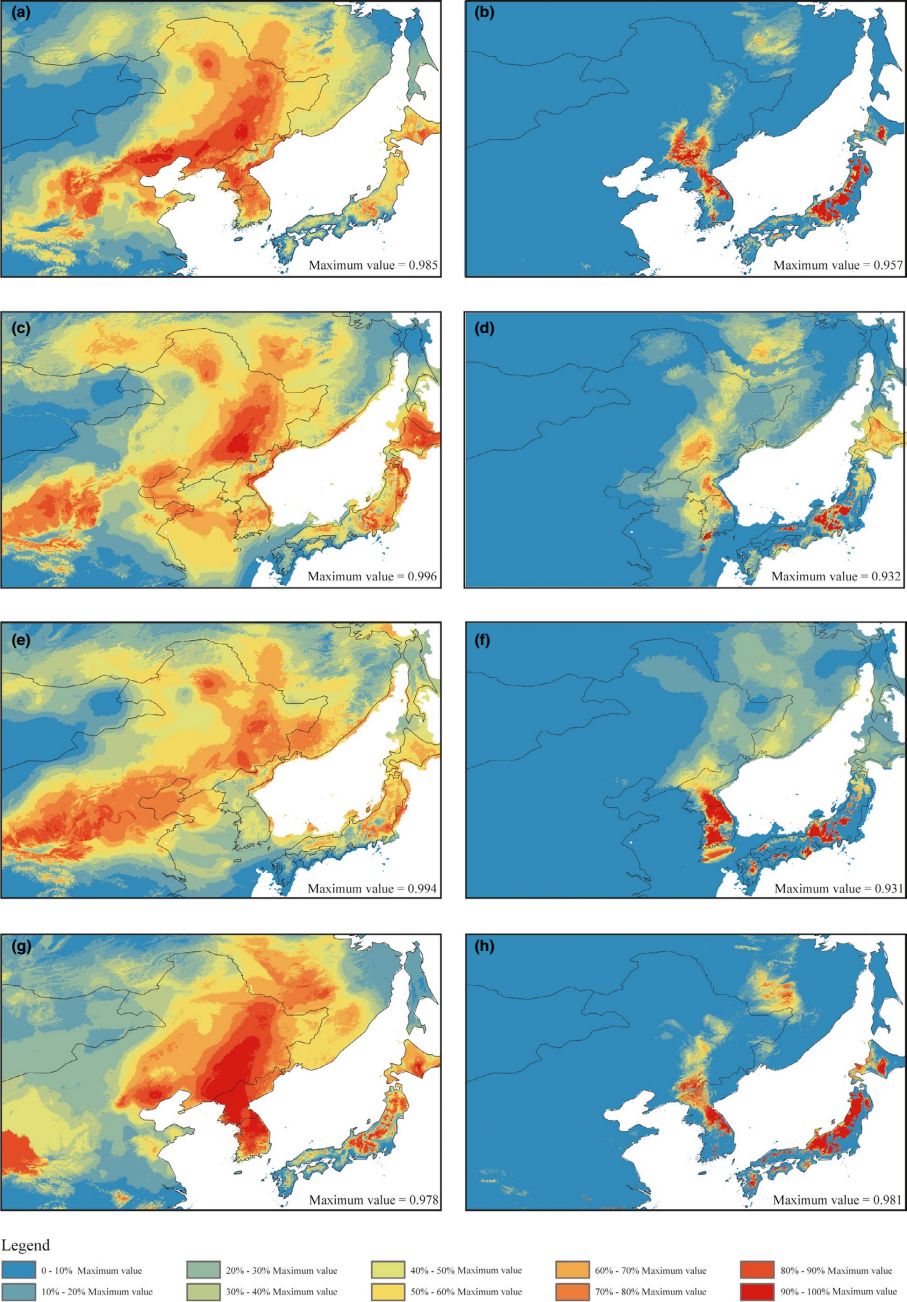
Modeled climatically suitable areas for *Betula platyphylla* (left) and *B. ermanii* (right). The present (a, b) and last glacial maximum (LGM: c. 21 Ka BP) under the CCSM (c, d) and MIROC models (e, f). The last interglacial (LIG: c. 130 Ka BP) (g, h). The logistical value of habitat suitability is shown according to the color‐scale bars

We employed ecological niche models to infer the historical and potential distributions of *B. platyphylla* and *B. ermanii* (Figure 4). The two *Betula* species exhibited current distribution ranges in NEC predicted by the niche model, with an average area under the curve of 0.956 for *B. platyphylla* and 0.927 for *B. ermanii* (Figure 4a,b). These results correspond well to the extant ranges of *B. platyphylla* but are smaller than that of *B. ermanii*. Moreover, the predicted distributions of the two species during the LIG are similar to their current distributions (Figure 4g,h). The distribution range of the two *B. platyphylla* appears to be wider at present than during the LGM under both models (CCSM and MIROC). For *B. platyphylla*, the LGM model (Figure 4c,e) indicated two geographically separate areas in NEC with high suitability value regions (>0.8): the Changbai and Daxing'anling mountains. In contrast, the predicted distribution ranges for *B. ermanii* during the LGM were southward (Figure 4d,f).

## 4 DISCUSSION

Previous studies have provided a framework for understanding the impacts of the LGM on current terrestrial ecosystems (Clark et al., 2009; Comes & Kadereit, 1998; Hewitt, 1999, 2004). In the present study, we investigated the genetic structure of two *Betula* species based on multiple nuclear and chloroplast markers. The distinct genetic structures of the two species indicated different demographic histories between them.

### 4.1 Genetic diversity in *B. platyphylla* versus *B. ermanii*

In our study, only two nucleotide substitutions were detected in cpDNA sequences (Table S4). This may be because the chloroplast genome has a lower mutation rate than does the nuclear genome. We propose that only a few chloroplast haplotypes survived in the refuge together with repeated expansions and contractions during the Quaternary climatic oscillation. Of course, the low genetic diversity in the two species could simply be a result of low sampling, with an average of five samples from each population, though with random sampling within populations. However, 1 and 7 haplotypes were detected among *B. platyphylla* and *B. ermanii* populations sampled in Japan, and the *B. ermanii* population was mostly dominated by three haplotypes (Tsuda & Ide, 2010). Extremely low regional genetic variation is commonly found, particularly in the northern portion of the range of temperate species. For example, *Acer mono*, *Juglans mandshurica*, and *Eleutherococcus sessiliflorus* also show low cpDNA diversity in NEC (Bai, Wang, & Zhang, 2016; Guo et al., 2014; Wang, Bao, Wang, Wang, & Ge, 2016). We therefore believe that the result is not simply an artifact but due to historical events. Indeed, simultaneous cpDNA haplotype extinction events in NEC provide evidence that many of these northern populations may have experienced a bottleneck during the LGM (Hewitt, 2004, 2011).

Ar and Ht values, representing genetic diversity, were higher in *B. ermanii* than in *B. platyphylla* populations (Table S1). The former was expected to have higher genetic diversity than the latter because it is a tetraploid species. If *B. ermanii* were found to be an allotetraploid species, supported by clones of an individual, distributed in two clades (Figure S3), the level of allelic richness would be expected to be higher than that of a diploid species (*B. platyphylla*) because new alleles would derive by allopolyploidization. However, we cannot rule out the possibility of combined effects of autopolyploidy and admixture. We also suggest that the relatively high Ar and Ht of *B. ermanii* may be attributed to relatively broad LGM distributions and greater population connectivity in glacial and postglacial landscapes. Both gene diversity and allelic richness are generally higher in tetraploid birch species than in diploids (Tsuda et al., 2016).

The FIS values of nine populations of *B. platyphylla* and all populations of *B. ermanii* were significantly greater than zero (Table S1), showing a deficiency of heterozygotes relative to Hardy–Weinberg expectations. For *B. platyphylla*, no geographic pattern in the distributions of FIS values was observed, which might be due to the small sample size of individuals (*n* = 10) per population we used (Lobato‐de Magalhães, Cabrera‐Toledo, & Martínez, 2019; Meirmans & Van Tienderen, 2013). Previous studies in diploid birch species have shown that HWE can generally be assumed (Rusanen, Vakkari, & Blom, 2003; Tsuda & Ide, 2005; Tsuda et al., 2015, 2009). Polyploids, especially allopolyploids, always display higher values of FIS, confirming nonrecombinant inheritance of parental genomes (Efimov, Philippov, & Krivenko, 2016; Lobato‐de Magalhães et al., 2019). Accordingly, it was not beyond the expectation that the *B. ermanii* populations have higher FIS values.

### 4.2 Different genetic structures in *B. platyphylla *versus* B. ermanii*

Our study showed a slight range contraction during the LGM for both species (Figure 4). Microsatellite data identified divergent lineages of *B. platyphylla* in NEC, indicating the existence of cryptic refuge (Figure 3b,c). Furthermore, comparisons of NST and GST revealed a strong phylogeographic structure, and NST was significantly larger than GST for *B. platyphylla* (NST = 0.308, GST = 0.224, *p* < .05). Geographically separated populations of *B. platyphylla* exhibited high levels of genetic differentiation (online data), which may have been caused by current and historical isolation (Byrne et al., 2016). Rather than retreating southward, we inferred that the taxa might have taken refuge in their distributional range, such as in the Changbai Mountains. Previous phylogeographic studies on cool temperate deciduous trees also suggest in situ survival, such as for *Ostryopsis davidiana* (Tian et al., 2009), *Mongolian oak* (Zeng et al., 2015), and *Juglans mandshurica* (Bai et al., 2010). Although introgression may also explain this pattern and hybrid zones between *B. pendula* and *B. platyphylla* were detected in Siberia, we can still ignore this situation because no introgression in NEC was detected based on eighteen nSSRs or seven nSSRs in the study of Tsuda et al. (2016).

In contrast, no geographic separation of the nSSR variation in *B. ermanii* was found, nor was it observed for cpDNA (NST = 0.441, GST = 0.429, *p* > .05). Different genetic variation patterns were found between the two congeneric species *B. platyphylla* and *B. ermanii*, and a possible reason is that the two species inhabit two contrasting niches that led to a distinct history during and after the LGM. More cold‐tolerant *B. ermanii* was likely better able than *B. platyphylla* to survive at higher latitudes. It has been proposed that according to the pollen record, East Asia was not covered by an extensive terrestrial ice cap but was most likely covered by tundra and taiga forest during the LGM (Cao, Herzschuh, Ni, Zhao, & Böhmer, 2015; Qiu et al., 2011). Thus, a relative lack of geographic structure for *B. ermanii* in NEC may have resulted from high levels of gene flow between the geographically separated populations in the LGM. A similar result was observed in the phylogeographic study of four forest‐dependent bat species (Kuo, Chen, Fang, Flanders, & Rossiter, 2014). Although this study was not designed to collect samples from Japan, South Korea, and North Korea, the results do exclude the possibility of recolonization from one refuge because no difference in genetic diversity among the populations and low gene flow due to the isolation of distribution were found. In previous studies, no evidence of phylogeographic structure was detected in *B. ermanii* from other regions (Tsuda et al., 2016, 2009).

### 4.3 Multiple glacial refuges for *B. platyphylla* in NEC

In NEC, a total of 2,621 species were recorded, belonging to 128 families and 74 genera (Fu, 2003). In our study, the Changbai flora (northern Changbai Mountains) exhibited the greatest number of plant species (1,798) among the four floras in NEC (Figure 1). It is worth noting that the Greater Khingan flora also contains many plant species (1,497). The highest endemic species richness was also found in this area (19.9% for the Changbai flora and 15% for the Greater Khingan flora). High species richness and differences in species richness allowed us to propose a distinct demographic history in NEC. As Svenning (Svenning & Skov, 2007) found that the LGM climate constituted the best single set of explanatory variables for the richness of endemic species in Europe, we hypothesized that the taxa in NEC derived from at least two refuges.

To confirm this hypothesis, we performed population genetic analyses for two woody species, *B. platyphylla* and *B. ermanii*, based on nSSRs, nuclear, and chloroplast markers. As expected, populations of *B. platyphylla* collected from the Daxing'anling Mountains might have migrated from a cryptic refuge distinct from the Changbai Mountain populations. Theoretically, populations from different refuge would be expected to carry different private alleles, mainly due to long‐term isolation (Provan & Bennett, 2008). Indeed, a large proportion of private alleles based on seven nSSR loci were found in populations sampled from both the Daxing'anling Mountains and Changbai Mountains. Notably, the Xiaoxing'anling populations were found to exhibit a mixed population structure and share alleles with populations from both the Daxing'anling Mountains and Changbai Mountains, suggesting admixture colonization of the Xiaoxing'anling populations by distinct lineages following postglacial expansion.

We also simulated the historical and current distribution ranges of the two *Betula* species. Our results based on both CCSM and MIROC models predicted the existence of multiple glacial refuges in the Changbai and Greater Khingan mountains, indicating that *B. platyphylla* survived among both floras during the LGM. Our findings support the view that multiple LGM refuges may have allowed cool temperate species to persist in NEC (Bai et al., 2010; Zeng et al., 2015) and that the Daxing'anling Mountains might constitute another glacial refuge in NEC. Of course, the populations of white birch distributed in the Daxing'anling Mountains may also have recolonized from Siberia. Additional materials are needed to verify this hypothesis in future work.

## 5 CONCLUSIONS

Climatic oscillations during the Quaternary have led to the establishment of current terrestrial ecosystems. The high species richness and endemic species richness in the Changbai and Greater Khingan mountains suggest that these two regions may have different demographic histories. Here, phylogenetic analyses based on multiple chloroplast and nuclear genome markers demonstrate that populations of *B. platyphylla* in NEC had recolonized from two independent glacial refuges. More cold‐tolerant species were likely able to survive at higher latitudes, leading to high levels of gene flow between geographically separated populations during the LGM, with no geographic structure detected for *B. ermanii*.

## CONFLICTS OF INTEREST

The authors declare that they have no competing interests.

## AUTHORS CONTRIBUTION

HYW, XY, DXY, and LL conducted the experimental work and performed the analyses. HYW and XY drafted the manuscript. HXX participated in the design and coordination of this study, collected the samples, and helped draft the manuscript. All authors read and approved the final manuscript.
