# Supplementary material for: Population genetic structures of two ecologically distinct species Betula platyphylla and B. ermanii inferred based on nuclear and chloroplast DNA markers
Source: Ecol Evol. 2019 Sep 10;9(19):11406–19. doi: 10.1002/ece3.5643 (PMC6802015; doi:10.1002/ece3.5643)
Supplement: Supplementary file 1 [file ECE3-9-11406-s001.docx]

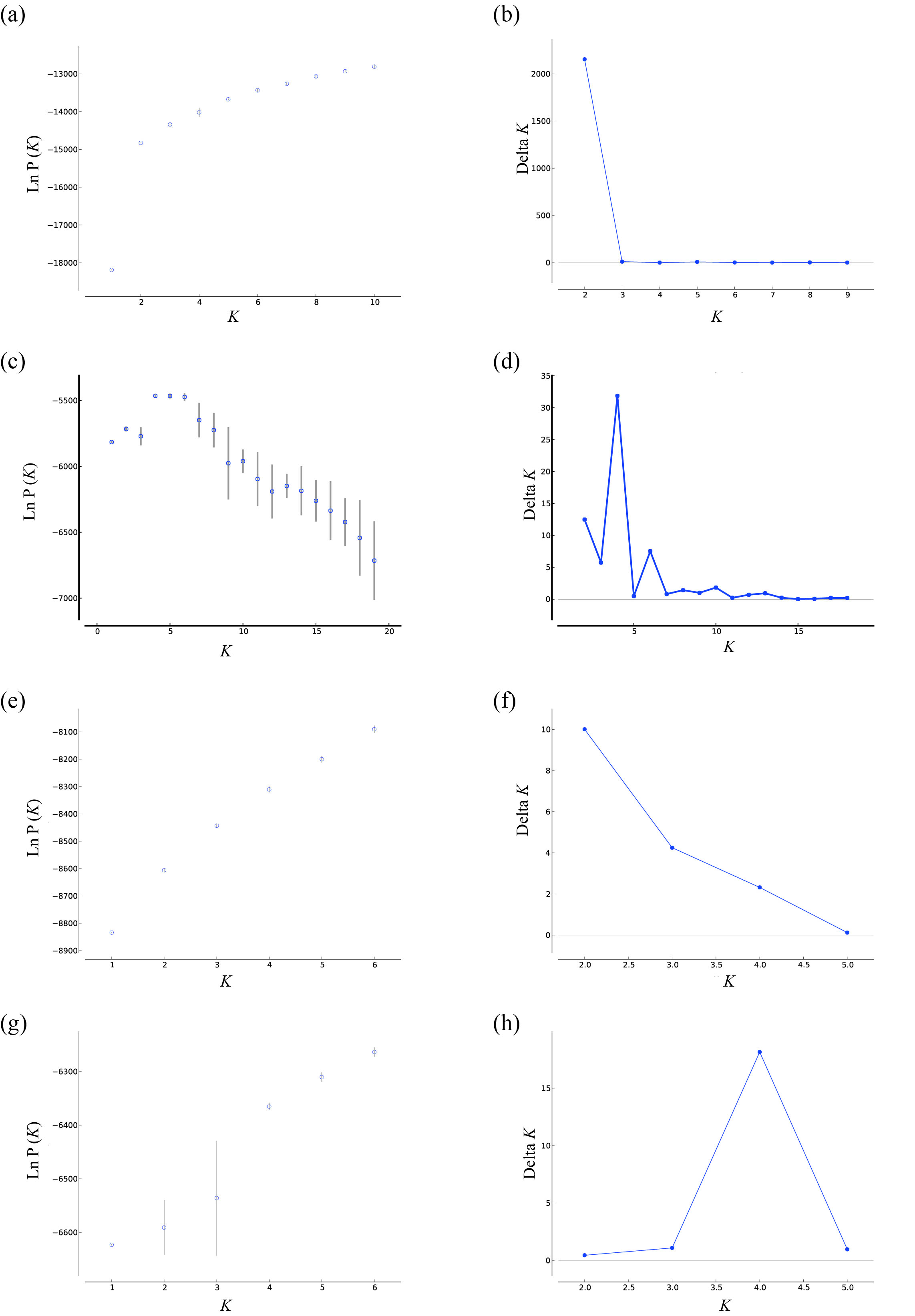


**Figure S1.** Maen Ln P(K) values (left) and ΔK (right) from Structure analysis of two species (a,b), *Betula platyphylla* (c, d), *B. ermanii* dataset with our manual estimates of allele copy numbers (e, f) and *B. ermanii* dataset with missing data (g, h).


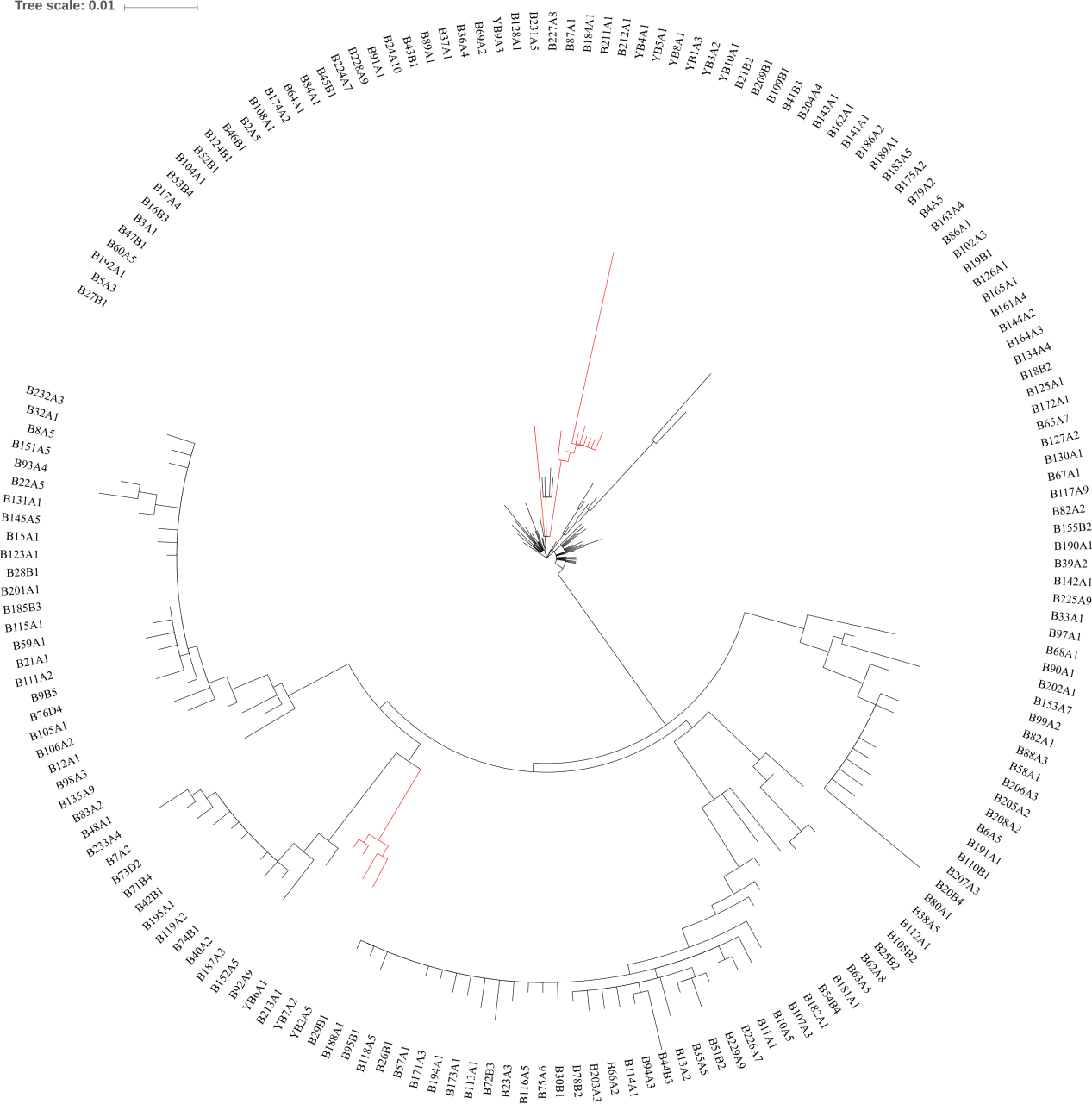


**Figure S2.** ML tree based on *G3PDH* in *Betula platyphylla*. Individuals from Korea are shown in red.

**
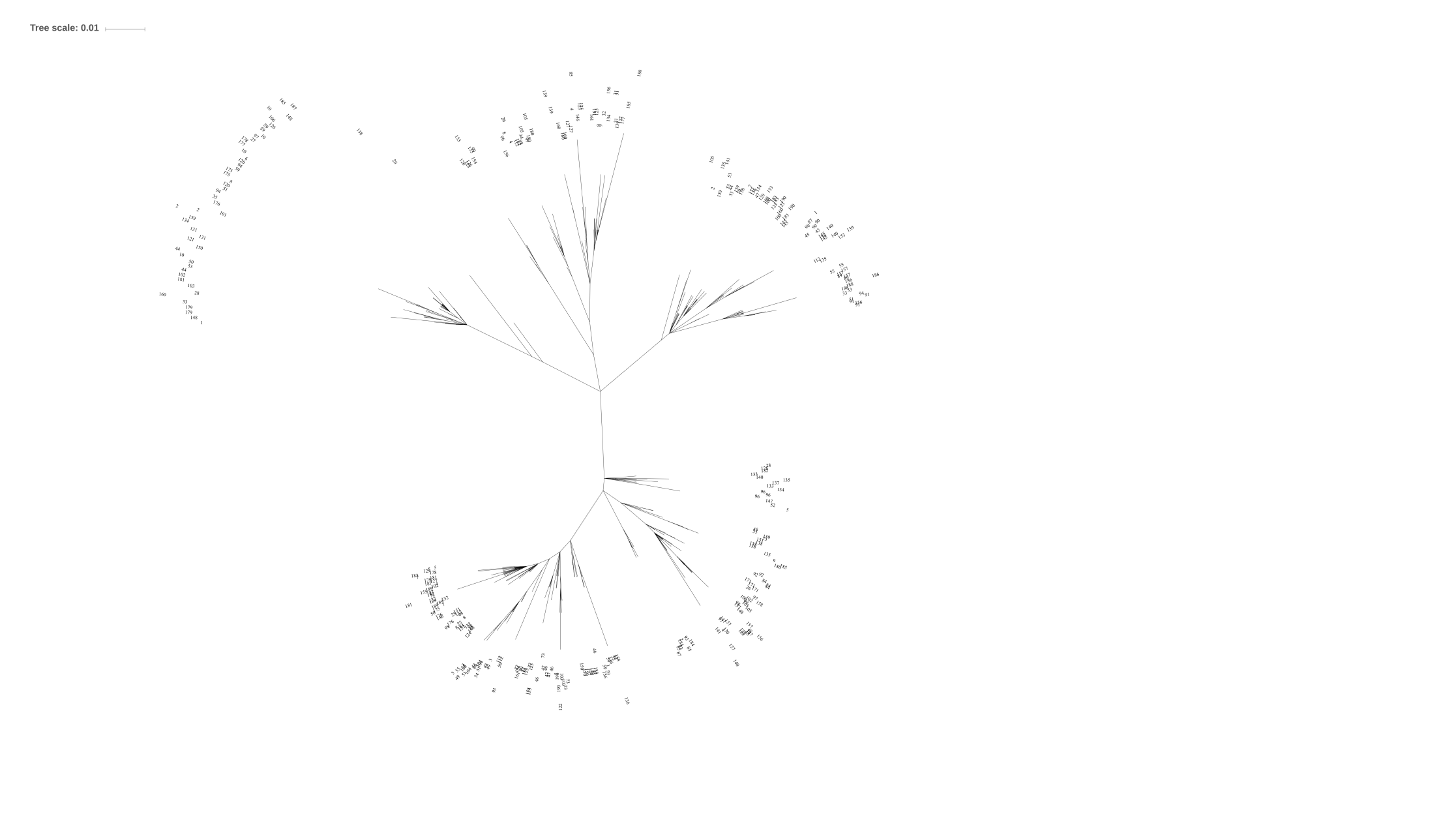
**

**Figure S3.** ML tree based on *G3PDH* in *B. ermanii*.

**Table S1.** Genetic diversity of two *Betula* species.

| Population name | Longitude (E) | Latitude (N) | cp | nSSR | | | | | | | nuDNA | | | | | | | | | |
| --- | --- | --- | --- | --- | --- | --- | --- | --- | --- | --- | --- | --- | --- | --- | --- | --- | --- | --- | --- | --- |
|  |  |  | size | size | N_A_ | N_P_ | Ar | Ho | Ht | Fis | size | clone | h | Hd | S | θ | π | D | D* | F* |
| BPJL | 126°19' | 42°11' | 5 | 12 | 45 | 0.03 | 3.82 | 0.531 | 0.642 | 0.173* | 9 | 9 | 9 | 1 | 73 | 0.035 | 0.0269 | -1.228 | -1.0418 | -1.2213 |
| BPJD | 127°45' | 41°26' | 5 | 10 | 35 | 0 | 3.75 | 0.513 | 0.681 | 0.247* | 8 | 8 | 8 | 1 | 60 | 0.030 | 0.0338 | 0.5517 | 0.3619 | 0.4534 |
| BPWHL | 127°52' | 43°29' | 5 | 11 | 37 | 0 | 3.92 | 0.557 | 0.653 | 0.147 | 10 | 10 | 10 | 1 | 82 | 0.040 | 0.0356 | -0.5849 | -0.8507 | -0.8862 |
| BPHW | 127°18' | 44°39' | 5 | 9 | 35 | 0 | 3.82 | 0.5 | 0.71 | 0.296* | 8 | 8 | 9 | 1 | 88 | 0.045 | 0.0418 | -0.3724 | 0.1444 | 0.0212 |
| BPHQ | 128 °33' | 44°24' | 4 | 6 | 25 | 0 | 3.51 | 0.521 | 0.625 | 0.167 | 6 | 7 | 7 | 1 | 73 | 0.040 | 0.0418 | 0.2176 | 0.1072 | 0.1462 |
| Changbai Mountains |  |  | 24 | 48 | 63 | 0.01 | 8.75 | 0.526 | 0.67 | 0.214* | 41 | 42 | 41 | 0.999 | 147 | 0.050 | 0.0377 | -0.7411 | -2.6200* | -2.313 |
| BPHS | 127°30' | 45°16' | 5 | 10 | 34 | 0.01 | 3.56 | 0.5 | 0.641 | 0.220* | 10 | 10 | 10 | 1 | 78 | 0.038 | 0.0405 | 0.285 | -0.0829 | 0.0104 |
| BPHZ | 132°52' | 45°26' | 5 | 10 | 33 | 0 | 3.78 | 0.438 | 0.628 | 0.303* | 9 | 9 | 9 | 1 | 81 | 0.040 | 0.0408 | 0.0628 | -0.3678 | -0.2958 |
| BPHX | 132°53' | 45°20' | 5 | 10 | 38 | 0 | 3.55 | 0.513 | 0.622 | 0.175 | 9 | 9 | 8 | 0.972 | 65 | 0.032 | 0.0273 | -0.7816 | -0.7629 | -0.8621 |
| BPHL | 129°53' | 45°46' | 4 | 5 | 26 | 0 | 3.63 | 0.525 | 0.675 | 0.222 | 4 | 4 | 4 | 1 | 9 | 0.006 | 0.0056 | -0.8294 | -0.8294 | -0.8234 |
| BPHP | 128°33' | 46°39' | 4 | 4 | 26 | 0.02 | 3.88 | 0.688 | 0.688 | 0 | 4 | 4 | 4 | 1 | 41 | 0.030 | 0.0294 | -0.261 | -0.1855 | -0.2108 |
| Xiaoxing’anling region |  |  | 23 | 39 | 60 | 0 | 9.75 | 0.51 | 0.655 | 0.221* | 36 | 36 | 35 | 0.998 | 122 | 0.042 | 0.0355 | -0.4343 | -7.6580* | -5.6924* |
| BPHWD | 126°04' | 48°41' | 5 | 10 | 38 | 0 | 3.78 | 0.537 | 0.663 | 0.190* | 9 | 9 | 9 | 1 | 86 | 0.042 | 0.0436 | 0.1369 | -0.1841 | -0.194 |
| BPHH | 127°24' | 50°14' | 5 | - | - | - | - | - | - | - | 9 | 9 | 9 | 1 | 80 | 0.041 | 0.0402 | -0.1517 | -0.0309 | -0.0677 |
| BPIK | 122°02' | 51°50' | 5 | 10 | 31 | 0.01 | 3.29 | 0.488 | 0.59 | 0.173 | 8 | 8 | 8 | 1 | 73 | 0.038 | 0.0336 | -0.6587 | -0.7823 | -0.8409 |
| BPIT | 122°23' | 50°35' | 5 | 10 | 34 | 0 | 3.44 | 0.512 | 0.562 | 0.088 | 8 | 8 | 8 | 1 | 80 | 0.044 | 0.039 | -0.6197 | -0.8365 | -0.8772 |
| BPIE | 123°32' | 50°33' | 5 | 10 | 43 | 0.04 | 4.05 | 0.588 | 0.694 | 0.153 | 10 | 11 | 8 | 0.096 | 79 | 0.038 | 0.0398 | 0.1822 | 0.3532 | 0.3508 |
| BPIY | 122°55' | 49°17' | 5 | 10 | 37 | 0.02 | 3.66 | 0.475 | 0.634 | 0.251* | 9 | 9 | 9 | 1 | 80 | 0.042 | 0.0404 | -0.2159 | -0.2959 | -0.3107 |
| BPIA | 120°06' | 47°13' | 5 | 10 | 38 | 0.04 | 3.64 | 0.562 | 0.662 | 0.151 | 8 | 8 | 7 | 0.964 | 78 | 0.039 | 0.0409 | 0.1719 | -0.1265 | -0.0632 |
| BPAW | 120°03' | 46°40' | - | - | - | - | - | - | - | - | 8 | 8 | 9 | 1 | 57 | 0.027 | 0.0282 | 0.2099 | 0.1801 | 0.2104 |
| BPIC | 118°46' | 41°53' | 4 | 9 | 45 | 0.05 | 4.1 | 0.528 | 0.697 | 0.243* | 8 | 9 | 9 | 1 | 63 | 0.031 | 0.0313 | 0.0984 | -0.1555 | -0.1055 |
| Daxing’anling region |  |  | 39 | 69 | 81 | 0.04 | 10.6 | 0.527 | 0.657 | 0.198* | 77 | 79 | 69 | 0.993 | 171 | 0.052 | 0.0342 | -1.1386 | -4.0155* | -3.3928* |
| NEC region |  |  | 87 | 156 | 95 | - | 10.1 | 0.522 | 0.689 | 0.241* | 154 | 157 | 136 | 0.996 | 249 | 0.072 | 0.0335 | -1.6605* | -7.1870* | -5.4767* |
| BPQL | 108°49'" | 33°50' | 5 | 8 | 35 | 0.01 | 3 | 0.391 | 0.586 | 0.333* | 8 | 8 | 7 | 0.964 | 77 | 0.039 | 0.0304 | -1.1513 | -1.3234 | -1.4323 |
| BPHG | 127°49' | 37°54' | 7 | 15 | 31 | 0.06 | 3.32 | 0.642 | 0.667 | 0.038 | 14 | 14 | 15 | 1 | 74 | 0.03 | 0.0266 | -0.4747 | -0.9785 | -0.9654 |
| Total |  |  | 98 | 179 | 101 | 0.20 | 14.6 | 0.527 | 0.648 | 0.186* | 176 | 179 | 154 | 0.996 | 282 | 0.081 | 0.034 | -1.7906* | -7.3979* | -5.5912* |
| BELL | 124°53' | 41°19' | 9 | 14 | 47 | 0.01 | 7.11 | 0.522 | 0.728 | 0.293* | 10 | 32 | 28 | 0.994 | 68 | 0.026 | 0.0196 | -0.9564 | -1.2979 | -1.4003* |
| BEJC | 128°03' | 42°03' | 11 | 34 | 67 | 0.01 | 8.48 | 0.585 | 0.712 | 0.182* | 33 | 110 | 90 | 0.995 | 176 | 0.056 | 0.0233 | -1.8838* | -4.1148* | -3.7602* |
| BELB | 127°55' | 44°14' | 13 | 30 | 62 | 0 | 8.05 | 0.52 | 0.697 | 0.259* | 29 | 92 | 80 | 0.996 | 144 | 0.047 | 0.0234 | -1.6465 | -4.0619* | -3.6476* |
| Changbai Mountains |  |  | 33 | 78 | 79 | 0.02 | 9.68 | 0.549 | 0.715 | 0.232* | 72 | 234 | 183 | 0.996 | 247 | 0.076 | 0.0219 | -2.1773* | -6.2692* | -5.0240* |
| BEHP | 128°33' | 46°39' | 10 | 22 | 62 | 0 | 8.19 | 0.493 | 0.704 | 0.308* | 3 | 15 | 13 | 0.981 | 48 | 0.022 | 0.0189 | -0.6317 | 0.5304 | 0.2376 |
| Xiaoxing’anling region |  |  | 10 | 22 | 62 | 0 | 9.44 | 0.493 | 0.704 | 0.301* | 3 | 15 | 13 | 0.981 | 48 | 0.022 | 0.0189 | -0.6317 | 0.5304 | 0.2376 |
| BEIK | 122°02' | 51°50' | 11 | 20 | 68 | 0.02 | 8.56 | 0.564 | 0.691 | 0.190* | 17 | 50 | 40 | 0.99 | 92 | 0.034 | 0.0228 | -1.1441 | -2.3581* | -2.2786* |
| BEIA | 120°06' | 47°13' | 10 | 16 | 59 | 0.03 | 7.83 | 0.559 | 0.686 | 0.193* | 16 | 52 | 49 | 0.998 | 121 | 0.042 | 0.0242 | -1.4866* | -3.1263* | -3.0005* |
| Daxing’anling region |  |  | 21 | 36 | 82 | 0.03 | 10.4 | 0.563 | 0.704 | 0.200* | 33 | 102 | 85 | 0.995 | 155 | 0.051 | 0.0238 | -1.7352* | -5.0415* | -4.337* |
| Total |  |  | 64 | 136 | 100 | 0.17 | 13.6 | 0.535 | 0.708 | 0.232* | 108 | 351 | 258 | 0.996 | 296 | 0.091 | 0.0221 | -2.2608* | -7.2972* | -5.4588* |

N_A_, N_P_: total number of alleles and number of private alleles per population at 7 nSSR markers;

h, number of haplotypes; Hd, haplotype diversity; S, number of segregating sites; θ, Watterson estimator of per base pair (Watterson, 1975);

π, average number of nucleotide differences per site between two sequences;

D, Tajima’s D (Tajima, 1983); D^a^, Fu and Li’s D; F^a^, Fu and Li’s F; *, *p* value < 0.05.

**Table S2.** Primer pairs of 40 chloroplast loci during the pre-experimental stage.

| Chloroplast regions | Primer names and sequences (5’ – 3’) | Tm (℃) | References |
| --- | --- | --- | --- |
| ***trnL^(UAA)^-trnF^(GAA)^*** | TrnL-F(f): ATTTGAACTGGTGACACGAG | 46 | Taberlet et al., 1991 |
|  | TrnL-F(c): CGAAATCGGTAGACGCTACG |  |  |
| ***trnH-psbA*** | psbA: CGAAGCTCCATCTACAAATGG | 47 | Hamilton, 1999 |
|  | trnH: ACTGCCTTGATCCACTTGGC |  |  |
| ***psbK-psbI*** | psbK: TTAGCCTTTGTTTGGCAAG | 48 | Lahaye et al., 2008 |
|  | psbI: AGAGTTTGAGAGTAAGCAT |  |  |
| *psbB-psbF* | psbB: GTTTACTTTTGGGCATGCTTCG | 50 | Hamilton, 1999 |
|  | psbFr: CGCAGTTCGTCTTGGACCAG |  |  |
| *trnS-trnG* | trnS(GCU): GCCGCTTTAGTCCACTCAGC | 52 | Hamilton, 1999 |
|  | trnG(UCC): GAACGAATCACACTTTTACCAC |  |  |
| *trnL^(UAA)^* | trnL-C: GGGGATAGAGGGACTTGAAC | - | Taberlet et al., 1991 |
|  | trnld: CGAAATCGGTAGACGCTACG |  |  |
| *trnT^(UGU)^-trnF^(GAA)^* | trnTf: CATTACAAATGCGATGCTCT | - | Taberlet et al., 1991 |
|  | trnFr: ATTTGAACTGGTGACACGAG |  |  |
| *rps16 intron* | rps16R2: TCGGGATCGAACATCAATTGCAAC | 48 | Oxelman et al., 1997 |
|  | rps16F: GTGGTAGAAAGCAACGTGCGACTT |  |  |
| *atpF-atpH* | atpF: ACTCGCACACACTCCCTTTCC | 46 | Lahaye et al., 2008 |
|  | atpH: GCTTTTATGGAAGCTTTAACAAT |  |  |
| *rpoC2* | rpoC2-f: CGAGCAGTTTCTTGRAAACTCGC | - | Grivet et al., 2001 |
|  | rpoC2-r: GAGGATTAATGKCRGATCCWCAAGG |  |  |
| *trnK* | trnK1: GGGTTGCCCGGGACTCGAAC | - | Grivet et al., 2001 |
|  | trnK2r: CAACGGTAGAGTACTCGGCTTTTA |  |  |
| *trnQ-trnS* | trnQf: GGGACGGAAGGATTCGAACC | 48 | Grivet et al., 2001 |
|  | trnS: ATTGCGTCCAATAGGATTTGAA |  |  |
| *trnC-trnD* | trnCf: CCAGTTCAAATCTGGGTGTC | - | Grivet et al., 2001 |
|  | trnDr: GGGATTGTAGTTCAATTGGT |  |  |
| *psbA-trnS* | psaAf: ACTTCTGGTTCCGGCGAACGAA | 47 | Grivet et al., 2001 |
|  | trnSr: AACCACTCGGCCATCTCTCCTA |  |  |
| *trnV-rbcL* | trnVf: CGAACCGTAGACCTTCTCGG | 48 | Grivet et al., 2001 |
|  | rbcLr: GCTTTAGTCTCTGTTTGTGG |  |  |
| *orf512-orf184* | orf512: AGTATGGGATCCGTAGTMGG | 48 | Grivet et al., 2001 |
|  | orf184r: GGCCYCGGATTTCCATATAAAG |  |  |
| *psbB1-psbB2* | psbB1: TGCCTTGGTATCGTGTTCATAC | 48 | Grivet et al., 2001 |
|  | psbB2r: CYTGTCTTYTTGTAGTTGGAT |  |  |
| *trnS-trnR* | trnS: CGCCGCTTTAGTCCACTCA | 48 | Grivet et al., 2001 |
|  | trnRr: ATTGCGTCCAATAGGATTTGAA |  |  |
| *rps18-clpp* | rps18f: GCTCGTATTTTATCTTTGTTACC | - | Grivet et al., 2001 |
|  | clppr: AACCTGCTAGTTCTTWTTAT |  |  |
| *rps8-rpl16* | rps8f: TGAACAATATTTTCGGTAAT | 47 | Grivet et al., 2001 |
|  | rpl16r: AACCAGATTTCGTAAACAAC |  |  |
| *atpH-atpI* | atpHf: CCAGCAGCAATAACGGAAGC | - | Grivet et al., 2001 |
|  | atpIr: ATAGGTGAATCCATGGAGGG |  |  |
| *atpI-rpoC2* | atpIf: GATGRCCCTCCATGGATTCACC | - | Grivet et al., 2001 |
|  | rpoC2r: GCGAGTTTTCAAGAAACTGCTCG |  |  |
| *clpp-psbB* | clppf: ATAAWAAGAACTAGCAGGTT | - | Grivet et al., 2001 |
|  | psbBr: ATAYACCCAATGCCARATAG |  |  |
| *trnD-trnT* | trnDf: ACCAATTGAACTACAATCCC | 48 | Grivet et al., 2001 |
|  | trnTr: CTACCACTGAGTTAAAAGGG |  |  |
| *psbC-trnS* | psbCf: GGTCGTGACCAAGAAACCAC | 50 | Grivet et al., 2001 |
|  | trnSr: GGTTCGAATCCCTCTCTCTC |  |  |
| *trnfM-psaA* | trnfMf: GAACCCGTGACCTCAAGGTTATG | 47 | Grivet et al., 2001 |
|  | psaAr: ATTCGTTCGCCGGAACCAGAAGT |  |  |
| *trnS-trnT* | trnSf: CGAGGGTTCGAATCCCTCTC | 46 | Grivet et al., 2001 |
|  | trnTr: AGAGCATCGCATTTGTAATG |  |  |
| *orf184-petA* | orf184f: GGCCYCGGATTTCCATATAAAG | 52 | Grivet et al., 2001 |
|  | petAr: CCCTCKGAAACAAGAAGTT |  |  |
| *petA-psbE* | petAf: GCATCTGTTATTTTGGCACA | - | Grivet et al., 2001 |
|  | psbEr: TACCTTCCCTATTCATTGCG |  |  |
| *psbL-rps18* | psbL: GAAAATAAAACAGCAAGTAC | - | Grivet et al., 2001 |
|  | rps18r: GGTAACAAAGATAAAATACGAGC |  |  |
| *psbB2-petB3* | psbB2f: CAGAAGCTTGGTCTAAAATTCC | - | Grivet et al., 2001 |
|  | petB3r: GRTCCCAAGGGAARGAATAACCAGT |  |  |
| *petD-rps8* | petDf: TCCAGTAGTAATTCTAGGTA | 48 | Grivet et al., 2001 |
|  | rps8r: TAAAATACTTTTACGAGAAG |  |  |
| *rps4-trnF^(GAA)^* | rps4R2: CTGTSAGACCATAATGAAAMCG | 48 | Shaw et al., 2005 |
|  | trnT^(UGU)^R: AGGTTAGAGCATCGCATTTG |  |  |
| *trnD^(GUC)^-trnT^(GGU)^* | trnT^(GCU)^: CTACCACTGAGTTAAAAGGG | 48 | Shaw et al., 2005 |
|  | trnE^(UUC)^R: TCCTTGAAAGAGAGATGTCCT |  |  |
| *ycf6-psbM* | psbM-RAq: ATGGAAGTAAATATTGTCGC | - | Shaw et al., 2005 |
|  | ycf6-FAq558: TCCATTGATTTGATTGTTTCG |  | Fior et al. 2013 |
| *ndhA intron* | ndhAx1: GCYCAATCWATTAGTTATGAAATACC | - | Shaw et al., 2007 |
|  | ndhAx2: GGTTGACGCCACAGATTCCA |  |  |
| *rpl32-trnL* | trnL^(UAG)^: CTGCTTCCTAAGAGCAGCGT | - | Shaw et al., 2007 |
|  | rpL32-F: CAGTTCCAAAAAAACGTACTTC |  |  |
| *ndhJ-trnF^(GAA)^* | ndhJ: ATGCCYGAAAGTTGGATAGG | 48 | Shaw et al., 2007 |
|  | TabFR: CTCGTGTCACCAGTTCAAAT |  |  |
| *psbJ-petA* | psbJ: ATAGGTACTGTARCYGGTATT | 47 | Shaw et al., 2007 |
|  | petA: AACARTTYGARAAGGTTCAATT |  |  |
| *trnK-matK* | trnK3914F: AAGATGTTGATWGTAAATGA | 50 | Johnson and Soltis, 1995 |
|  | matK1470R: TGGGTTGCTAACTCAATGG |  |  |

Tm, Temperature used in this study.

**Table S3.** Analysis of molecular variance (AMOVA) for two *Betula* species based on

seven nSSRs.

| Source of Variation | Total variance (%) | *F*-statistics | *p*-value |
| --- | --- | --- | --- |
| Within Population | 55.6 | Rho_ST_ = 0.444 | - |
| Among Population | 5.9 | Rho_SC_ = 0.096 | 0.001 |
| Among species | 38.5 | Rho_CT_ = 0.385 | 0.001 |

**Table S4.** Nucleotide variations in the chloroplast region of two *Betula* species.

| haplotype | 523-531 | 723-724 | 763 | 766 | 769 | 876-877 | 888-896 | 916 | 939 | 998-1000 | 1203 |
| --- | --- | --- | --- | --- | --- | --- | --- | --- | --- | --- | --- |
| H1 | ----ATTTT | T- | A | A | - | -T | TAATTAGTA | C | T | -TT | G |
| H2 | ----ATTTT | T- | A | A | - | -T | TAATTAGTA | T | T | -TT | G |
| H3 | ----ATTTT | T- | A | A | - | -T | TAATTAGTA | C | T | --T | G |
| H4 | ----ATTTT | T- | A | A | - | AA | TAATTAGTA | C | - | TTT | G |
| H5 | ----ATTTT | T- | A | A | - | -T | TAATTAGTA | C | T | --T | G |
| H6 | --------- | T- | A | A | - | -T | TAATTAGTA | C | T | --T | G |
| H7 | ----ATTTT | T- | A | A | - | -T | TAATTAGTA | C | T | -TT | G |
| H8 | ----ATTTT | T- | A | A | - | -T | TAATTAGTA | C | T | --T | G |
| H9 | TT--ATTTT | T- | A | A | - | -T | TAATTAGTA | C | T | --T | G |
| H10 | TT--ATTTT | T- | A | A | - | -T | TAATTAGTA | C | T | --- | G |
| H11 | TT--ATTTT | T- | A | A | - | -T | TAATTAGTA | C | T | --T | G |
| C1 | ----ATTTT | TT | A | A | - | -T | --------A | C | T | -TT | G |
| C2 | ----ATTTT | T- | A | A | - | -T | --------A | C | T | -TT | G |
| C3 | ----ATTTT | T- | A | A | G | -T | --------A | C | T | -TT | G |
| C4 | ----ATTTT | -- | A | A | - | -T | --------A | C | T | -TT | G |
| C5 | ----ATTTT | T- | - | A | - | -T | --------A | C | T | -TT | G |
| C6 | ----ATTTT | T- | A | A | - | -T | -------AA | C | T | -TT | G |
| C7 | T---ATTTT | T- | A | A | - | -T | --------A | C | T | -TT | G |
| C8 | T---ATTTT | T- | A | A | - | -T | --------A | C | T | -TT | T |
| C9 | TTTTATTTT | T- | A | A | - | -T | --------- | C | T | -TT | G |
| C10 | TTTTATTTT | TT | A | A | - | -T | --------- | C | T | -TT | G |
| C11 | TTTTATTTT | TT | A | - | - | -T | --------- | C | T | -TT | G |

**Table S5.** Diversity analysis of the two *Betula* species based on 7 nSSR markers*.*

|  | BP4 | | | BP12 | | | BP15 | | | BP16 | | | BP17 | | | BE6 | | | BE12 | | |
| --- | --- | --- | --- | --- | --- | --- | --- | --- | --- | --- | --- | --- | --- | --- | --- | --- | --- | --- | --- | --- | --- |
|  | NA | h | Ar | NA | h | Ar | NA | h | Ar | NA | h | Ar | NA | h | Ar | NA | h | Ar | NA | h | Ar |
| BPJL | 6 | 0.644 | 3.678 | 2 | 0.159 | 1.565 | 12 | 0.898 | 5.825 | 6 | 0.773 | 4.112 | 7 | 0.705 | 3.647 | 7 | 0.659 | 3.854 | 5 | 0.443 | 2.723 |
| BPJD | 8 | 0.800 | 4.809 | 2 | 0.100 | 1.400 | 8 | 0.861 | 5.024 | 6 | 0.794 | 4.064 | 6 | 0.739 | 4.165 | 5 | 0.778 | 3.715 | 3 | 0.583 | 2.398 |
| BPWHL | 8 | 0.886 | 5.411 | 1 | 0.000 | 1.000 | 11 | 0.923 | 6.126 | 4 | 0.482 | 2.818 | 8 | 0.745 | 4.140 | 5 | 0.614 | 3.051 | 5 | 0.700 | 3.469 |
| BPHW | 7 | 0.847 | 4.629 | 2 | 0.208 | 1.706 | 8 | 0.833 | 5.039 | 4 | 0.667 | 3.139 | 5 | 0.785 | 3.970 | 4 | 0.757 | 3.361 | 4 | 0.701 | 3.146 |
| BPHQ | 4 | 0.717 | 3.558 | 2 | 0.167 | 1.667 | 6 | 0.750 | 4.576 | 4 | 0.633 | 3.315 | 6 | 0.683 | 4.333 | 3 | 0.767 | 2.980 | 2 | 0.433 | 1.982 |
| BPHS | 8 | 0.700 | 4.305 | 2 | 0.189 | 1.653 | 6 | 0.811 | 4.353 | 6 | 0.639 | 3.758 | 5 | 0.528 | 3.105 | 5 | 0.767 | 3.694 | 4 | 0.683 | 3.183 |
| BPHZ | 4 | 0.700 | 3.408 | 2 | 0.100 | 1.400 | 9 | 0.911 | 5.721 | 2 | 0.344 | 1.898 | 8 | 0.711 | 4.207 | 7 | 0.756 | 4.312 | 4 | 0.567 | 2.950 |
| BPHX | 7 | 0.583 | 3.653 | 2 | 0.267 | 1.807 | 7 | 0.844 | 4.898 | 4 | 0.433 | 2.705 | 5 | 0.572 | 3.098 | 7 | 0.850 | 4.697 | 5 | 0.722 | 3.757 |
| BPHL | 6 | 0.900 | 5.200 | 2 | 0.200 | 1.800 | 4 | 0.800 | 3.778 | 3 | 0.375 | 2.600 | 5 | 0.775 | 4.378 | 4 | 0.750 | 3.756 | 3 | 0.800 | 3.000 |
| BPHP | 4 | 0.750 | 4.000 | 2 | 0.250 | 2.000 | 5 | 0.917 | 5.000 | 4 | 0.625 | 4.000 | 4 | 0.750 | 4.000 | 4 | 0.708 | 4.000 | 3 | 0.667 | 3.000 |
| BPHWD | 5 | 0.572 | 3.098 | 2 | 0.400 | 1.949 | 7 | 0.744 | 4.410 | 6 | 0.517 | 3.253 | 8 | 0.794 | 4.711 | 6 | 0.833 | 4.430 | 4 | 0.567 | 2.950 |
| BPIK | 3 | 0.472 | 2.349 | 2 | 0.189 | 1.653 | 7 | 0.867 | 5.039 | 5 | 0.617 | 3.350 | 7 | 0.739 | 4.150 | 4 | 0.678 | 3.047 | 3 | 0.422 | 2.298 |
| BPIT | 5 | 0.439 | 2.853 | 2 | 0.100 | 1.400 | 7 | 0.833 | 4.814 | 5 | 0.578 | 3.358 | 8 | 0.811 | 4.799 | 5 | 0.750 | 3.677 | 3 | 0.194 | 1.800 |
| BPIE | 7 | 0.767 | 4.402 | 2 | 0.189 | 1.653 | 8 | 0.806 | 4.711 | 7 | 0.639 | 3.905 | 10 | 0.861 | 5.404 | 6 | 0.789 | 4.043 | 5 | 0.678 | 3.505 |
| BPIY | 6 | 0.728 | 4.003 | 2 | 0.189 | 1.653 | 9 | 0.933 | 5.859 | 5 | 0.439 | 2.853 | 5 | 0.761 | 3.832 | 6 | 0.661 | 3.549 | 4 | 0.589 | 3.112 |
| BPIA | 9 | 0.883 | 5.432 | 2 | 0.333 | 1.898 | 8 | 0.906 | 5.510 | 4 | 0.439 | 2.705 | 5 | 0.717 | 3.757 | 5 | 0.794 | 3.980 | 4 | 0.650 | 3.047 |
| BPIC | 8 | 0.764 | 4.634 | 4 | 0.396 | 2.595 | 10 | 0.924 | 6.010 | 4 | 0.396 | 2.595 | 8 | 0.882 | 5.239 | 6 | 0.743 | 3.970 | 4 | 0.653 | 2.885 |
| BPQL | 4 | 0.705 | 3.393 | 1 | 0.000 | 1.000 | 5 | 0.759 | 4.066 | 3 | 0.241 | 2.000 | 4 | 0.821 | 3.758 | 4 | 0.732 | 3.361 | 4 | 0.786 | 3.445 |
| BPHG | 7 | 0.821 | 4.443 | 2 | 0.429 | 1.965 | 6 | 0.807 | 4.398 | 3 | 0.571 | 2.599 | 3 | 0.567 | 2.461 | 7 | 0.860 | 4.906 | 3 | 0.562 | 2.456 |
| BELL | 7 | 0.797 | 4.305 | 7 | 0.841 | 4.614 | 8 | 0.814 | 4.502 | 4 | 0.636 | 3.037 | 3 | 0.233 | 1.846 | 10 | 0.825 | 4.657 | 8 | 0.845 | 4.709 |
| BEJC | 8 | 0.779 | 4.130 | 10 | 0.845 | 4.895 | 11 | 0.797 | 4.354 | 8 | 0.724 | 3.533 | 3 | 0.140 | 1.527 | 11 | 0.831 | 4.838 | 16 | 0.882 | 5.365 |
| BELB | 7 | 0.769 | 4.024 | 11 | 0.855 | 5.046 | 9 | 0.818 | 4.535 | 6 | 0.615 | 2.814 | 3 | 0.066 | 1.259 | 13 | 0.831 | 4.870 | 13 | 0.908 | 5.781 |
| BEHP | 8 | 0.754 | 3.766 | 12 | 0.869 | 5.296 | 9 | 0.788 | 4.358 | 6 | 0.745 | 3.641 | 3 | 0.067 | 1.265 | 14 | 0.838 | 4.911 | 11 | 0.869 | 5.193 |
| BEIK | 11 | 0.826 | 4.724 | 12 | 0.849 | 5.001 | 11 | 0.809 | 4.685 | 8 | 0.745 | 3.879 | 1 | 0.000 | 1.000 | 16 | 0.876 | 5.419 | 10 | 0.850 | 5.038 |
| BEIA | 9 | 0.794 | 4.256 | 10 | 0.858 | 5.017 | 11 | 0.854 | 4.989 | 8 | 0.727 | 3.995 | 2 | 0.063 | 1.243 | 8 | 0.771 | 4.016 | 9 | 0.690 | 3.998 |
| total | 18 | - | 5.150 | 15 | - | 4.394 | 24 | - | 5.524 | 15 | - | 3.819 | 22 | - | 3.712 | 24 | - | 4.691 | 19 | - | 5.336 |
